# Atmospheric Transport Towards the Iberian Peninsula in the 3- to 10-Day Range

**DOI:** 10.1100/tsw.2006.208

**Published:** 2006-08-25

**Authors:** Raquel Nieto, Luis Gimeno

**Affiliations:** ^1^ Universidad de Vigo, Facultad de Ciencias de Ourense, 32004, Ourense, Spain; ^2^ CGUL Departamento de Fisica, Faculdade de Ciencias, Universidade de Lisboa, Lisboa, Portugal

**Keywords:** sources of middle-lived pollutants, sources of aerosols, Lagrangian atmospheric transport model, Iberian Peninsula

## Abstract

An advanced Lagrangian atmospheric transport model (FLEXTRAP) was used to identify the possible sources of middle-lived pollutants over the Iberian Peninsula. A period of 4 years, 2000—2003, was analyzed. Transatlantic transport is the main pathway of the air reaching the Iberian Peninsula in the studied range of 3—10 days; local sources are limited to 3 days of transport. The presence of North America as a source from days 6—10 of transport identifies this region as the main potential contributor to the middle-lived pollutants over the Iberian Peninsula.

## INTRODUCTION

Atmospheric transport on timescales of the order of 3–10 days is crucial for substances that have a lifetime within this range. This involves many different substances, such as ozone ($O_3$) and its precursors, aerosols, mercury, or persistent organic pollutants. During the last years, different research projects around the world have performed field campaigns along the Iberian coasts, measuring air pollutants in the troposphere. Some of them evidenced that the formation and distribution of primary pollutants in urban plumes, at regional or continental scales, in the boundary layer and in the free troposphere, are linked together[1,2]. Parts of these studies are based to take into account the smaller scale in meteorology[3]. However, the aim of this work is to estimate possible sources of pollutants at intercontinental scales, so the micro- and mesoscale are not as important in this study. Due to the westerly direction of dominant winds in extratropical latitudes, the most probable source of Spanish pollutants at an intercontinental scale should be North America. However, there are several studies that point out that pollutants from North America are hardly ever observed at ground sites in Europe[4,5,6,7,8]. Depending on the meteorological situation, some type of pollution layer can be transported all the way to Europe[9]. From a dynamic point of view, the major processes responsible for vertical uplifting of polluted air masses to higher altitudes over the U.S. are synoptic-scale warm conveyor belts (WCBs) associated with frontal systems[5,10,11,12,13,14]. Pollutants can easily enter into the jet stream and be transported rapidly to Europe, where they are transported to the mid-upper troposphere. Europe frequently receives the outflow from WCBs that originate along the eastern North American coast[13,Stohl (2001)]. The period selected in this work (3–10 days) has the typical timescale of a WCB transport[14].

New advanced Lagrangian atmospheric transport models enable us to establish, almost unambiguously, source-receptor relationships over long distances that correspond to 10 days of transport. The study reported herein aims at characterizing the pathways for these middle-lived substances arriving in the Iberian Peninsula by using the successful Lagrangian particle dispersion model FLEXPART[15] and meteorological analysis data from the European Centre for Medium-Range Weather Forecasts (ECMWF)[16].

The FLEXPART model is a recognized tool to study physical processes from surface to upper troposphere, such as airstreams (cold conveyor belts, warm conveyor belts, dry intrusions, stratosphere-troposphere flows, and troposphere-to-stratosphere flows)[17,18]. The model resolves well the atmospheric systems that connect different sublayers of the troposphere (e.g., boundary layer, middle troposphere, uppermost troposphere) and the stratosphere.

## METHOD

As in Stohl et al.[15], the atmosphere was “filled” homogeneously into a large number of so-called particles, each representing a fraction of the total atmospheric mass, and then these particles were transported by the FLEXPART model using three-dimensional winds, with their positions being recorded every 6 h. In the work reported here, we used the tracks of 1,398,801 particles over a 4-year period (2000–2003). We tracked trajectories backwards from an area covering the Iberian Peninsula (Fig. 1), limiting the transport times to 10 days.

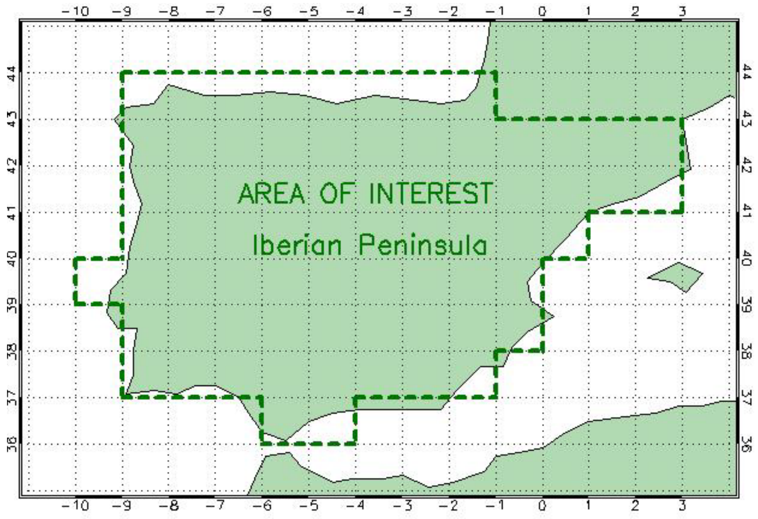

**FIGURE 1.** Map of the analyzed region (Iberian Peninsula).

Full details of the FLEXPART model can be found in Stohl et al.[7]; however, we summarize now the main characteristics. The FLEXPART model computed ECMWF operational analysis every 6 h (at 00, 06, 12, and 18 UTC) with a $1^{o}\times 1^{o}$ resolution on 60 vertical levels. There are approximately 14 model levels below 1500 m and 23 below 5000 m. To ensure exact mass balance, vertical winds are calculated using spherical harmonics data as part of the data-retrieval procedures at ECMWF. In order to account for turbulence, the FLEXPART model calculates the trajectory of the particles using analyzed winds plus random motions. In the planetary boundary layer (PBL), these random motions are calculated by solving

Langevin equations for Gaussian turbulence[18]. These equations use the Lagrangian timescales and the standard deviations of the wind components, which are computed from ECMWF PBL parameters[19]. The PBL height is diagnosed using a combined Richardson number and lifting parcel technique[20], and outside the PBL, turbulence is assumed to be very small. Global datasets also do not resolve individual convective cells, although they reproduce the large-scale effects of convection. FLEXPART has different options for how particles are generated and what they represent. In this case, the atmosphere was "filled" homogeneously with particles, each representing a fraction of the total atmospheric mass. Particles were then allowed to move freely (forward in time, but this is arbitrary) with the winds for the duration of the simulation.

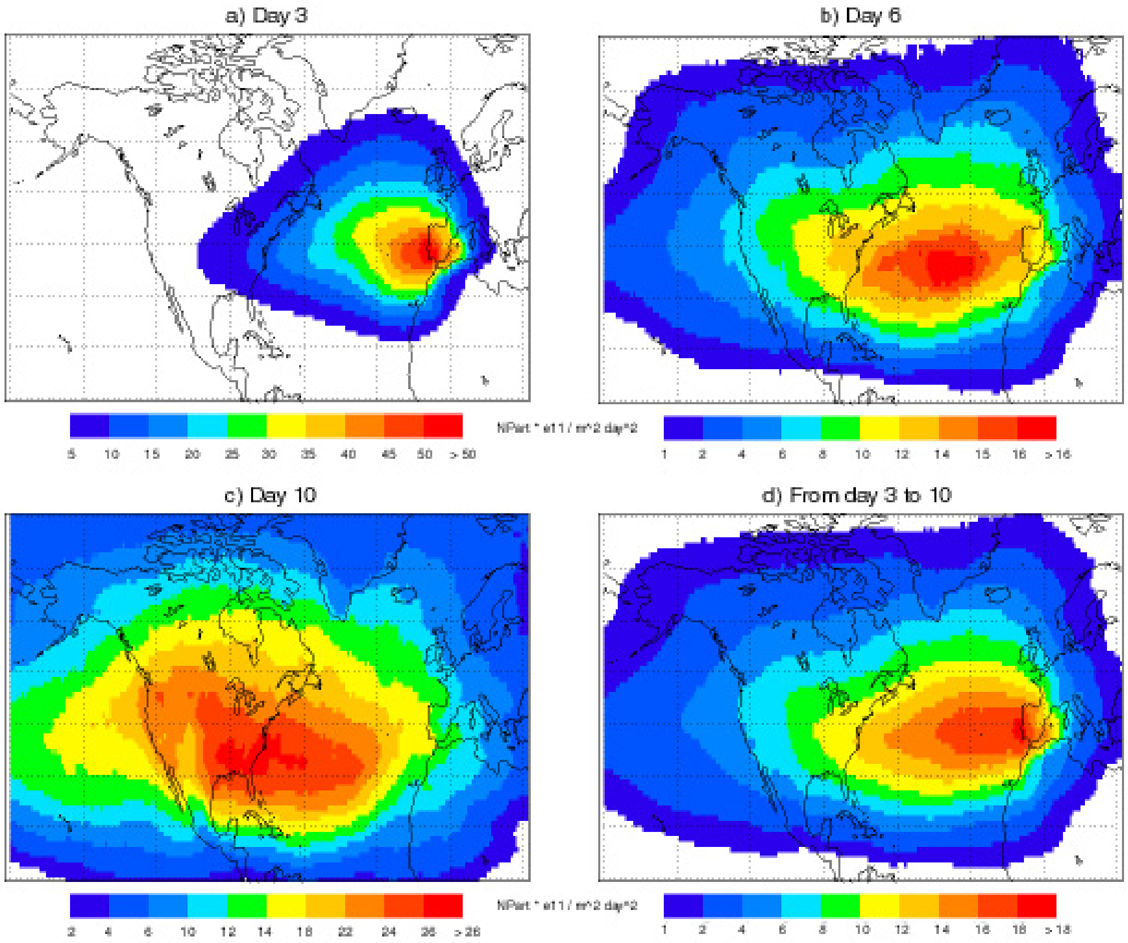

**FIGURE 2.** Annually averaged number of particles reaching the Iberian Peninsula that reside over a source area for (a) 3 days back, (b) 6 days back, (c) 10 days back, and (d) averaged over days 3–10 back (units are number of particles × $10^{11}$ $m^{-2}$ $day^{-2}$).

## RESULTS

We tracked the air masses residing over the whole column over the Iberian Peninsula back in time to determine their source. Fig. 2 shows the number of particles residing over a place (source) on the 3$^{rd}$, 6$^{th}$, and 10$^{th}$ days of transport and averaged over days 3–10 for the whole 4-year period (01.01.2000 to 12.31.2003). Three days back in time, most of the air resided over the Atlantic Ocean, with a maximum of residence on the Atlantic coast of the Iberian Peninsula. The source pattern is elongated westward because of the dominant westerly circulation and because the zonal transport is faster than the meridional transport in the extratropical Northern Hemisphere. As expected, the influence of the Mediterranean Sea, central Europe, and the British Islands is much less important than in the case of tracking the first 2 days of transport (which includes aerosols and especially short-lived pollutants[21,22]). Similar source regions can be observed for the 6$^{th}$ day of transport, with a logical expansion mainly to the west reaching North America. The displacement westwards continues in the 10$^{th}$ day of transport, reaching most of North America with the main source region being over the southeast Atlantic coast. The finding that North America is the main source of intercontinental pollution is in clear agreement with the general circulation of the atmosphere with dominant westerlies in extratropical latitudes[23] and with previous studies of pollutant transport (see, for example, examination of the transport of emissions from boreal forest fires in Canada that caused a dense layer of haze over Germany in August 1998[24]). The local influence (with the Iberian Peninsula itself as a source) is negligible for both the 6$^{th}$ and 10$^{th}$ days of transport. The average transport over days 3–10 shows that the origin of most of the air reaching the Iberian Peninsula in that period is an extratropical elongated area from the Middle Mediterranean to the Pacific American coasts, with the Atlantic Ocean being the main source.

Another interesting possibility with this Lagrangian method is to provide a quantification of the number of particles in transport. More precisely, it is appealing to evaluate the relative weight of the main source region, Eastern North America, if we compare it with regions like Central Europe and Northern Africa. We have quantified the number of particles series calculated backwards from the Iberian Peninsula and integrated over these three regions (Fig. 3). Fig. 3 shows the limits of the source regions (top) and depicts the values of the number of particles without considering the different areas of each source region (bottom). A view of Fig. 3 (bottom) shows that Eastern North America is clearly the most important source up to 3–10 days back. The supply of particles from Northern Africa or Central Europe is less than the supply from Eastern North America, however during the 3$^{rd}$ day, the contribution is similar between Europe and North America.

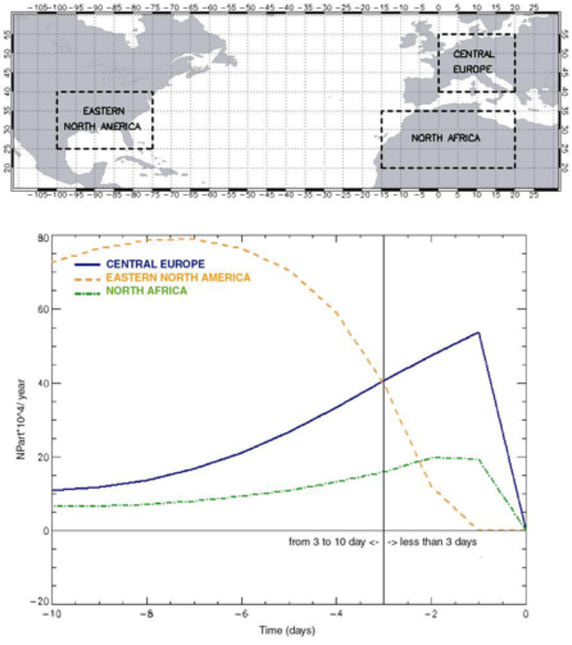

**FIGURE 3**. Time series of the number of particles calculated backward over the Iberian Peninsula area and integrated over the regions indicated in figure. (Top) Central Europe (blue), Eastern North America (orange), and Northern Africa (green); (bottom) absolute values of the number of particles time series (scale regulated by a factor of 10,000).

These patterns of particle residence were very robust, so similar structures appeared when the analysis was done with a seasonal base. Fig. 4 shows the seasonal number of residing particles averaged over days 3–10 of transport. The seasonal differences with the annual pattern are limited to a higher expansion to the west of the source areas for seasons with stronger winds, mainly during winter and autumn when the baroclinic activity is higher.

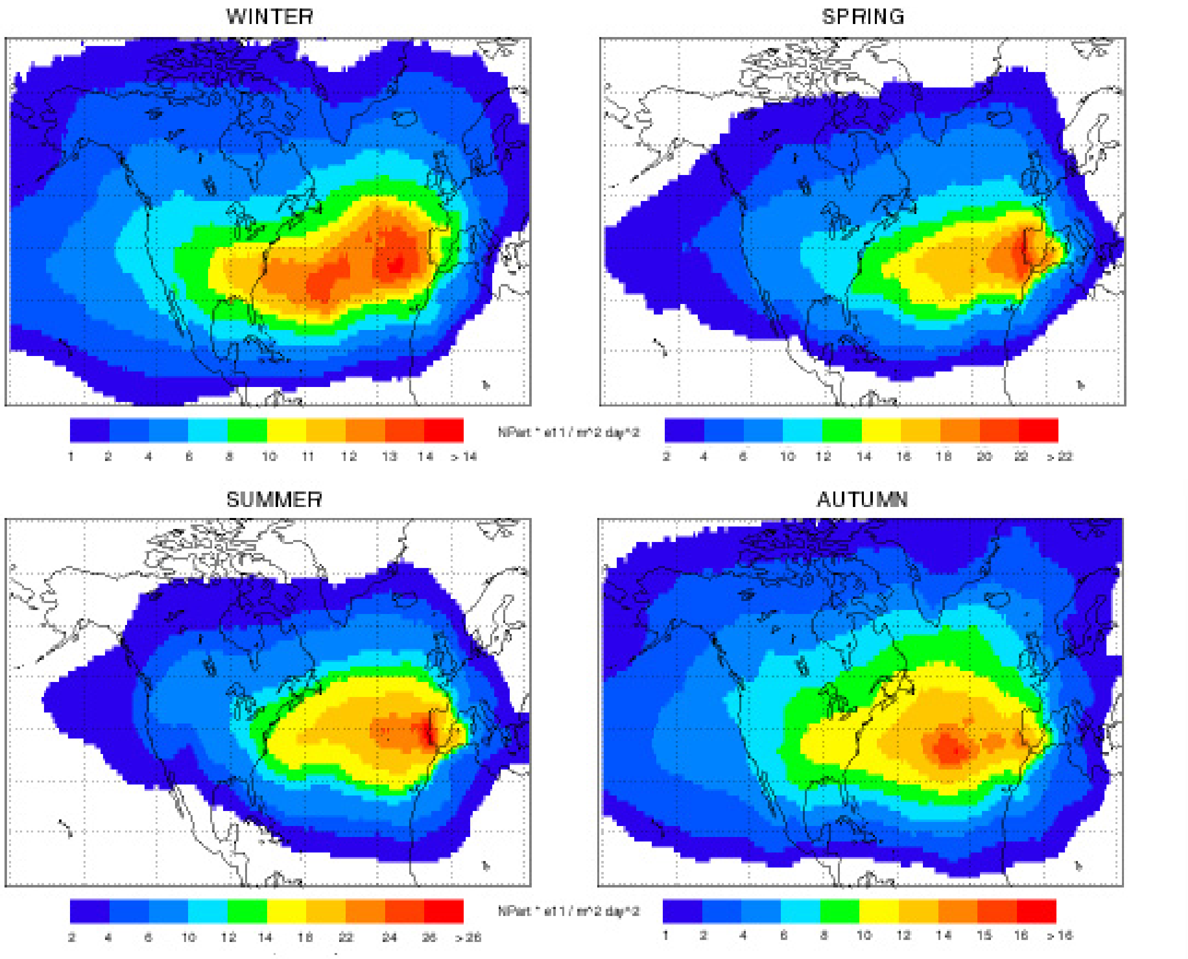

**FIGURE 4**. Seasonally averaged number of particles reaching the Iberian Peninsula that reside over a source area, averaged over days 3–10 back (units are number of particles × $10^{11}$ $m^{-2}$ $day^{-2}$).

## CONCLUDING REMARKS

Three conclusions can be extracted from this study:

1. Transatlantic transport is the main pathway of the air reaching the Iberian Peninsula in the studied range of 3–10 days.
2. The importance of local sources is limited to the 3rd day of transport.
3. The pattern of residence of particles during the 10th day of transport confirms the clear influence of North American sources over the Iberian Peninsula.

These conclusions are in agreement with previous studies of the intercontinental transport of pollutants and aerosols. Due to the strong anthropogenic-produced aerosols and pollutant emissions in the eastern half of North America[25], this region is the main potential contributor to the middle-lived pollutants over the Iberian Peninsula advected from outside.

## ACKNOWLEDGMENTS

We thank Andreas Stohl for providing the trajectory data and the Xunta de Galicia, the FCT of Portuguese Ministry of Science (SFRH/BPD/22178/2005), and the Spanish Ministry of Education and Science for granting the stays of R. Nieto and L. Gimeno in the Centro de Geofísica da Universidade de Lisboa through the programmes Bolsas para estadias no estranxeiro and Programa Nacional de ayudas para la movilidad de profesores de universidad e investigadores españoles y extranjeros.

This study was supported by grant "CGL2004-05187-C03-02" from the Spanish Ministry of Education and Science.
